# Porcine ovarian cortex-derived putative stem cells can differentiate into endothelial cells in vitro

**DOI:** 10.1007/s00418-021-02016-6

**Published:** 2021-07-16

**Authors:** Kamil Wartalski, Gabriela Gorczyca, Jerzy Wiater, Zbigniew Tabarowski, Małgorzata Duda

**Affiliations:** 1 Faculty of Medicine, Department of Histology, Jagiellonian University Medical College, Kopernika 7 Street, 31-034 Krakow, Poland; 2 Faculty of Biology, Institute of Zoology and Biomedical Research, Department of Endocrinology, Jagiellonian University in Krakow, Gronostajowa 9 Street, 30-387 Krakow, Poland; 3 Faculty of Biology, Institute of Zoology and Biomedical Research, Department of Experimental Hematology, Jagiellonian University in Krakow, Gronostajowa 9 Street, 30-387 Krakow, Poland

**Keywords:** Putative stem cells, Cell differentiation, Endothelial cells, Ovary, Pig

## Abstract

Endothelial cells (ECs), the primary component of the vasculature, play a crucial role in neovascularization. However, the number of endogenous ECs is inadequate for both experimental purposes and clinical applications. Porcine ovarian putative stem cells (poPSCs), although not pluripotent, are characterized by great plasticity. Therefore, this study aimed to investigate whether poPSCs have the potential to differentiate into cells of endothelial lineage. poPSCs were immunomagnetically isolated from postnatal pig ovaries based on the presence of SSEA-4 protein. Expression of mesenchymal stem cells (MSCs) markers after pre-culture, both at the level of mRNA: *ITGB1, THY,* and *ENG* and corresponding protein: CD29, CD90, and CD105 were significantly higher compared to the control ovarian cortex cells. To differentiate poPSCs into ECs, inducing medium containing vascular endothelial growth factor (VEGF), basic fibroblast growth factor (bFGF), insulin-like growth factor (IGF), epidermal growth factor (EGF), ascorbic acid, and heparin was applied. After 14 days, poPSC differentiation into ECs was confirmed by immunofluorescence staining for vascular endothelial cadherin (VECad) and vascular endothelial growth factor receptor-2 (VEGFR-2). Semi-quantitative WB analysis of these proteins confirmed their high abundance. Additionally, qRT-PCR showed that mRNA expression of corresponding marker genes: *CDH5, KDR* was significantly higher compared with undifferentiated poPSCs. Finally, EC functional status was confirmed by the migration test that revealed that they were capable of positive chemotaxis, while tube formation assay demonstrated their ability to develop capillary networks. In conclusion, our results provided evidence that poPSCs may constitute the MSC population in the ovary and confirmed that they might be a potential source of ECs for tissue engineering.

## Introduction

Regenerative medicine offers several promising solutions in the development of cures of some untreatable diseases ranging from diabetes mellitus and heart infarct to Parkinson's disease and premature ovarian failure. In this aspect, stem cell application, including adult stem cells (ASCs), seems to be one of the most promising therapeutic strategies. ASCs are rare tissue-specific cells of the postnatal organism into which they are committed to differentiate (Grompe 2002; Raff 2003). ASCs have been identified in many mammalian organs and tissues, including bone marrow, peripheral blood, skeletal muscle, skin, gut, brain, or testis (National Institutes of Health—NIH, USA 2016), in specific areas of each tissue, known as a stem cell niche (Morrison and Spradling 2008).

The role of ASCs lies in maintaining, generating, and replacing terminally differentiated cells within their specific tissue lost due to physiological cell turnover or tissue damage caused by injury (Weissman 2000). Recent data suggest that ASCs have the potential to generate differentiated cells even beyond their tissue boundaries, which is referred to as developmental plasticity (Galliot and Ghila 2010; Catacchio et al. 2013). Some reports also prove that in the same tissue, e.g. in the skin, both multipotent and unipotent populations of ASCs can exist (Wagers and Weissman 2004). Moreover, based on the expression of some marker genes, such as Oct-4 or Nanog, and the ability of a population of ASCs to differentiate into various cell types, ASC pluripotency is also postulated (Jiang et al. 2002; D'Ippolito et al. 2004).

Until recently, the mammalian ovary was considered a completely differentiated organ. Several studies conducted in recent years, also by our team, have provided evidence for the presence of ASCs in mammalian ovaries, due to their high diversity also referred to as putative stem cells (PSCs) (Wartalski et al. 2016; Yazdekhasti et al. 2017; Patel et al. 2018). Our latest results (Wartalski et al. 2020) demonstrated that porcine ovarian putative stem cells (poPSCs) isolated from the ovarian cortex, despite expression of pluripotency markers, are not pluripotent. As poPSCs can differentiate into functional neural-like cells, they may be multipotent, and poPSCs likely represent mesenchymal stem cell (MSC) populations within the ovary. In physiological conditions, PSCs can regulate ovarian functions and, in particular, regenerate them to some extent. This is important because at ovulation, oocyte release from the ruptured ovarian follicle causes damage to the ovarian surface. Therefore, PSCs can participate in the process of its regeneration (Stimpfel et al. 2014). However, full phenotypic characterization and understanding of all possible differentiation pathways of PSCs is still needed.

The promising therapeutic nature of MSCs is widely indicated, and they are being used to reconstruct damaged tissues in various pathological states (Baksh et al. 2004; Bianco et al. 2013). MSCs can be isolated, for example, from bone marrow, umbilical cord, adipose tissue, muscle or endometrium (Kern et al. 2006; Ceusters et al. 2017; Wiater et al. 2018). The diagnostic potential of MSCs lies in their ability for in vitro growth as a homogeneous population, adherence to plastic dishes, expression of a set of surface antigens that can be used for positive selection (e.g. CD105, CD90, CD29), lack of hematopoietic markers and the ability to differentiate in vitro into multiple cell lineages, including osteocytes, chondrocytes, adipocytes or skeletal muscle cells (Pittenger et al. 1999; Dominici et al. 2006). Moreover, multiple studies have shown that MSCs are ideal for in vitro generation of endothelial cells (ECs) for cell therapy (Ikhapoh et al. 2015; Quan et al. 2017; Tancharoen et al. 2017).

ECs line the interior surface of arteries, capillaries, veins, and lymphatic vessels with a thin, single layer of squamous cells. ECs regulate many critical physiological processes, including vasoconstriction and vasodilation, thereby controlling pressure and blood flow. Additionally, the endothelium balances the homeostasis between clotting and fibrinolysis. Disturbances in the proper functioning of the endothelium are common and dangerous. ECs may contribute to the development of diseases such as hypertension or coronary artery disease (Vanhoutte et al. 2017). Moreover, it is now increasingly recognized that dysfunctional ECs contribute to the development of nonvascular diseases such as neurodegenerative disorders (Koizumi et al. 2016) and chronic inflammatory disorders (Bordy et al. 2018) and, consequently, tumorigenesis (Dudley 2012). The endothelium is generally considered a dispersed, dynamic, and heterogeneous complex organ that forms, among others, the blood–brain barrier (Abbott et al. 2006).

For endothelial differentiation of poPSCs in our experiments we used growth factors which are usually applied to differentiate MSCs into ECs (Soleimani and Nadri 2009; Araña et al. 2013). These were vascular endothelial growth factor (VEGF), primary fibroblast growth factor (bFGF), epidermal growth factor (EGF), and insulin-like growth factor 1 (IGF-1). VEGF is especially active during embryonic development and angiogenesis. It occurs in several isoforms but all VEGF family proteins act through cell surface tyrosine kinase receptors, e.g. VEGFR-2 (Holmes et al. 2007). In turn, EGF is a factor commonly found in various tissues and organs (Hollenberg and Gregory 1980; Venturi and Venturi 2009). EGF, acting through its receptor, EGFR, influences the proliferation, differentiation, and survival of many types of cells (Herbst 2004). IGF-1 is one of the most potent natural activators of the protein kinase B (PKB) kinase signaling pathway. IGF-1 stimulates the growth and proliferation of cells and is also a potent inhibitor of apoptosis (Peruzzi et al 1999; Juin et al. 1999).

This study investigated two protein markers associated with differentiation into ECs: vascular endothelial cadherin (VECad) and VEGFR-2. VECad is a highly specific endothelium adhesion molecule, located at the junctions between ECs. VE-cadherin is responsible for the maintenance and control of EC contacts and plays an essential role during the morphogenesis of the vascular system. Mechanisms that regulate VE-cadherin-mediated adhesion are important for the control of vascular permeability and leukocyte extravasation (Corada et al. 2001). In addition to its adhesive functions, VE-cadherin regulates various cellular processes such as cell proliferation and apoptosis and modulates vascular endothelial growth factor receptor functions (Vestweber 2008). Of the three known VEGF receptors, VEGFR-2 is the primary regulator of EC proliferation and migration (Takahashi et al. 2005). VEGFR-2 is expressed in all human vascular ECs and is often overexpressed in highly malignant solid tumors (Smith et al. 2010).

Among various animal experimental models, pigs share many similarities to humans in the form of organ size, physiology, and functioning (Whyte and Prather 2011). The limited ethical dilemmas, successful ASCs isolation from different tissues (Wiater et al. 2018; Burian et al. 2017), and most importantly the rapid development of genetic engineering techniques enabling genome modifications in pigs that reduce the cross-species immune barrier (Hryhorowicz et al. 2017), make pigs the valuable experimental model for preclinical assessment of ASCs for stem cell therapy.

In light of the above, our research hypothesis assumed that the ovaries of sexually immature pigs contain multipotent PSCs of a mesenchymal nature that can also differentiate into ECs. If we could show that poPSCs, under very specific culture conditions, acquire particular features of the endothelium, poPSCs might be an alternative source for ECs for clinical therapies like tissue replacement or artificial organ vascularization. To meet this goal we examined (1) *ITGB1*, *THY*, *ENG*, *CDH5,* and *KDR* mRNA expression, and (2) CD29, CD90, CD105, VECad, and VEGFR-2 proteins abundance and immunolocalization in poPSCs and obtained by their differentiation endothelial-like cells. We also examined the functional status of such cells using the migration and tube formation assays.

## Materials and methods

### Sample collection and poPSC isolation

Porcine ovaries were excised from sexually immature Polish Landrace gilts (approximately 5–6 months of age and weighing 60–70 kg) at a local abattoir under veterinarian control within 10 min after slaughter. Next, they were placed in sterile ice-cold Dulbecco's phosphate-buffered saline (DPBS; pH 7.4, PAA, Cell Culture Company, Piscataway, NJ, USA) supplemented with antibiotics (Antibiotic/Antimycotic solution; AAS; 1% (v/v), PAA, Cell Culture Company) and transported to the laboratory within 1 h. After washing the experimental material twice using sterile DPBS, the ovarian cortex was separated from the ovarian cord with a scalpel and cut into uniform-size pieces of ~ 1 mm^3^ with a tissue slicer. The obtained fragments of ovarian cortex were subjected to a 2-h enzymatic digestion procedure in a Liberase™ TH Research Grade solution (0.26 U/mL in PBS; Sigma-Aldrich, St. Louis, MO, USA) in an incubator at 37 °C, with 150 rotations/min. Next, enzymatic digestion was terminated by adding an equal volume of cold DPBS (+4 °C) containing 10% fetal bovine serum (FBS). After that, the resulting suspension was filtered through 100-, 70- and 40-micron nylon strainers. In the further step, cells were washed several times in sterile DPBS and recovered by centrifugation (90×*g* for 10 min). poPSCs were isolated by an immunomagnetic method, modified and described by us previously (Wartalski et al. 2016), using a monoclonal antibody—anti-human SSEA-4, conjugated to magnetic beads (EasySep™ hESC/hiPSC SSEA-4 Positive Selection Kit, StemCell™ Technologies, Vancouver, Canada). Next, the poPSCs were cultured in the maintenance medium (MM): DMEM/F12 medium (Sigma-Aldrich) supplemented with 2% B-27 (Thermo Fisher Scientific, Waltham, MA, USA) and 2 μL/mL SCF (Thermo Fisher Scientific). The prepared suspension of 3 × 10^3^ cells/mL was seeded into the culture dishes. Cells for total protein or total RNA extraction after the experiment were cultured in six-well polystyrene plates (Nunc™, Thermo Fisher Scientific) coated with poly-l-lysine (Sigma-Aldrich). Cells for immunofluorescence studies were cultured on eight-cell Lab-Tek™ II—CC2 (Nunc™, Thermo Fisher Scientific) slides also coated with poly-l-lysine.

### poPSC SSEA-4^+^ culture and endothelial differentiation

The expansion of poPSCs was run according to a previously reported protocol, with certain modifications (Wartalski et al. 2020). In detail, the MM medium was changed every 2 days for freshness. Morphological properties of poPSCs were regularly checked by microscopic observation (inverted Nikon Ti-U microscope equipped with a Nikon DS-Fi1c-U3 camera, Tokyo, Japan) and analysis of proliferation (TC20 automated cell counter, Bio-Rad). After the third passage, when an appropriate number of poPSCs was obtained, they were divided into an uninduced group (the control cultures in the MM medium) and an induced group (the experimental cultures), where the MM medium was used to replace the commercial one: EGM™-2 (endothelial cell growth medium-2 BulletKit; Lonza, Basel, Switzerland). EGM™-2 is specifically designed for the culture of vascular ECs, as the kit includes, in addition to the medium, endothelial differentiation factors such as vascular endothelial growth factor (VEGF), basic fibroblast growth factor (bFGF), insulin-like growth factor (IGF), epidermal growth factor (EGF), ascorbic acid, and heparin. Cultures were carried out for 14 days at 37 °C in an atmosphere of 5% CO_2_ and 95% relative humidity. During this time, the medium (EGM™-2) was changed every 2 days, and cells were passaged when they reached 80% confluence. After completion of culture on day 14, total protein and total RNA were extracted from cells growing in six-well plates. Cells growing on eight-chamber slides were fixed for immunofluorescence. Some of the cells were left in culture for further migration and tube formation tests.

### Immunofluorescence

Immunofluorescence was conducted as described previously (Wartalski et al. 2020; Gorczyca et al. 2019). Briefly, after completion of the culture, cells were washed with DPBS and fixed with cold 4% paraformaldehyde in DPBS for 10 min. After several washes with DPBS, permeabilization of cell membranes was performed by using 0.1% Triton X-100 (Sigma–Aldrich) in Tris-buffered saline (TBS; pH 7.4). In the next step, non-specific binding sites were blocked by incubation with 5% normal goat serum (NGS, Sigma–Aldrich) in a humidity chamber for 40 min at room temperature (RT). After that, NGS was removed and cells were incubated with the primary antibodies raised against EC markers VECad (polyclonal rabbit anti-VECad, dilution: 1:100, Abcam, cat. #ab33168) and VEGFR-2 (monoclonal mouse anti-VEGFR-2, dilution: 1:50, Santa Cruz, cat. #sc-393163) overnight at 4° C in a humidity chamber. Antibodies used to confirm the presence of MSC markers in poPSC cells were as follows: (monoclonal rabbit anti-CD29, dilution: 1:100, Abcam, cat. #ab134179), (monoclonal mouse anti-CD90, dilution: 1:100, Abcam, cat. #ab134179), and (monoclonal mouse anti-CD105, dilution: 1:100, Abcam, cat. #ab134179). Antibodies used to confirm lack of CD45 as a negative marker of MSCs (polyclonal rabbit anti CD45, dilution: 1:100, Abcam, cat. # ab10559).

Next, cells were washed several times in TBST (TBS + 0.1% Tween 20, Sigma-Aldrich) and incubated with the appropriate secondary antibodies: goat anti-rabbit (Thermo Fisher Scientific, cat. #A11070) or goat anti-mouse (Thermo Fisher Scientific, cat. #A28175) IgG labeled with Alexa Fluor 488 diluted 1:500 for 1 h at RT in the dark, humidity chamber. Negative controls were performed by replacing the primary antibodies with appropriate, non-immune mouse (NI03, Sigma-Aldrich) or rabbit (NI01; Sigma-Aldrich) IgG. Rhodamine phalloidin (Invitrogen™ Thermo Fisher Scientific) was used to visualize the vascular-like structures formed during the tube formation test. Immunofluorescence-labeled cells were mounted in VectaShield^®^ HardSet™ Mounting Medium with DAPI (Vector Laboratories, Burlingame, CA, USA) and were analyzed with an Olympus FLUOVIEW FV1200 scanning confocal laser microscope using laser wavelengths and powers set for respective fluorochromes as indicated by their manufacturers.

### Western blot analysis

Total protein extraction and Western blot analysis were conducted as previously described (Wartalski et al. 2020). Protein content was examined using the Bradford method (Bradford 1976). Samples were separated by 12% SDS-PAGE under reducing conditions (Laemmli 1970) and electroblotted into a PVDF membrane employing a semi-dry blotter in Genie transfer buffer (pH 8.4). The blotted membranes were blocked for 1 h. at RT in TBST containing 5% non-fat dry milk followed by overnight incubation at +4 °C with primary antibodies (polyclonal rabbit antibody anti-VE-cadherin, dilution: 1: 1000, Abcam, cat.#ab33168; monoclonal mouse anti-VEGFR-2, dilution: 1:100, Santa Cruz, cat. #sc-393163; monoclonal rabbit antibody anti-CD29, dilution: 1:1000, Abcam, cat. #ab134179; monoclonal mouse antibody anti-CD90, dilution: 1:1000, Abcam, cat. #ab23894; monoclonal mouse antibody anti-CD105, dilution: 1:500, Abcam, cat. #ab44967)) and then with horseradish peroxidase-conjugated goat anti-rabbit IgG (dilution: 1:2000, Thermo Fisher Scientific cat. #31460) or goat anti-mouse IgG (dilution: 1:2000, Bio-Rad Laboratories cat. #1706516) secondary antibodies for 1.5 h at RT. Chemiluminescent signal was developed with WesternBright™ ECL Blotting Substrate (Advansta, Menlo Park, CA, USA) and visualized with the ChemiDoc™ XRS+ System (Bio-Rad Laboratories Inc., GmbH, Munchen, Germany).

After washing, each membrane was stripped and reprobed with monoclonal mouse anti–β-actin antibody (dilution: 1:2000, Sigma-Aldrich, cat. #A5441) which was used as a reference protein, followed by horseradish peroxidase-linked goat anti-mouse IgG (dilution 1:2000; Bio-Rad Laboratories cat. # 1706516). The bands were densitometrically quantified and normalized to their corresponding β-actin bands using the ImageLab 2.0 Software (Bio-Rad Laboratories Inc., Hercules, CA, USA). Western blot analysis was performed for three separately repeated experiments.

### RNA isolation, reverse transcription, and quantitative real-time polymerase chain reaction analysis

Total cellular RNA was extracted from poPSCs, ECs, ovarian cortex, porcine skin, and aorta (frozen in liquid nitrogen as a positive control) using EZ-10 Spin Column Total RNA Mini Preps Super Kit (Bio Basics, Canada Inc.) following the manufacturer’s protocol. The RNA quality and quantity were determined by the A260/A280 ratio using a NanoDrop™ Lite Spectrophotometer (Thermo Scientific, Wilmington, DE, USA) and the RNA integrity was evaluated through the observation of 18S and 28S ribosomal bands after electrophoresis on 1% formaldehyde-agarose gel.

An equivalent amount of total RNA (1 µg) for each sample was reverse-transcribed with the High-Capacity cDNA Reverse Transcription Kit (Applied Biosystems, Foster City, CA, USA) as previously described (Gorczyca et al. 2019). The resulting cDNA was used for quantitative PCR using the TaqMan Gene Expression Master Mix (Applied Biosystems) and porcine-specific TaqMan Gene Expression Assays (Applied Biosystems) for *ITGB1* (Ss03391118_m1), *THY1* (Ss03376963_u1), *ENG* (Ss03391353_m1), *CDH5* (Ss03378336_u1), and *KDR* (Ss03376639_u1) following manufacturers’ instructions. Glyceraldehyde-3-phosphate dehydrogenase (GAPDH, Ss03373286_u1) was employed as endogenous control. Real-time PCR reactions were performed in duplicate with StepOne™ Plus Real-Time PCR System (Applied Biosystems) according to the recommended cycling program (2 min at 50 °C, 10 min at 95 °C, 40 cycles of 15 s at 95 °C, and 1 min at 60 °C). Amplification of contaminating genomic DNA was checked by control experiments in which reverse transcriptase were omitted during the RT step. Threshold cycles (Ct values) for the expression of the investigated gene were calculated using StepOne software. All samples were normalized to GAPDH (^ΔΔCt^ value). The relative mRNA transcript abundance of genes of interest was expressed as 2^−∆∆Ct^ (Livak and Schmittgen 2001) and was used to calculate statistical differences.

### Functional analysis of poPSCs differentiated into endothelial cells by migration and tube formation tests

The transwell migration assay through a porous membrane was adapted based on the work by Justus et al. (2014). Briefly, after 14 days of poPSC differentiation into ECs, they were washed with DPBS without Ca^2+^ and Mg^2+^ ions and digested with 0.25% trypsin with EDTA (Sigma–Aldrich) as for passage procedure. Next, they were suspended in a serum-free medium and 1 × 10^6^ cells were seeded into transwell ThinCert™ inserts (Greiner Bio-One) with a bottom from a porous membrane made of PET (polyethylene terephthalate; a pore diameter of 3 µm and a density of 0.6 × 10^6^/cm^2^), which were placed in the culture wells of a 24-well plate. The lower chambers were filled with 600 μL of DMEM/F12 supplemented with 20% FBS as a chemoattractant or with the same volume of serum-free medium (a negative control). Cells were cultured in standard conditions (37 °C, in an atmosphere of 5% CO_2_ and 95% relative humidity) for 48 h. The non-migrating cells on the upper side of the inserts were removed with a cotton swab and those attached to the lower side were fixed with 70% ethanol and stained with 0.5% crystal violet. Polycarbonate membranes with stained cells were removed from transwell inserts and photographed in a Nikon Eclipse Ni–U microscope (Nikon).

The tube formation test was adapted for research based on the work of Nakatsu and Hughes (2008). In brief, after 14 days of poPSC differentiation into ECs, they were washed with DPBS without Ca^2+^ and Mg^2+^ ions and digested with 0.25% trypsin with EDTA as for passage procedure. Cytodex Microcarrier beads (C3275, Sigma-Aldrich) were hydrated in DPBS for at least 3 h at RT. After beads settlement, the supernatant was discarded and fresh Ca^2+^ and Mg^2+^ free DPBS was added to a stock concentration of 50 mL/g. The beads in DPBS were sterilized by autoclaving at 120 °C for 20 min and stored at +4 °C. To use, the bead suspension was mixed thoroughly, 1 mL was pipetted to a 15-mL conical centrifuge tube, and the mixture was centrifuged at 400 g for 5 min. The supernatant was carefully aspirated and microcarrier beads were re-suspended in a volume of 10 mL DMEM/F12 culture medium supplemented with 10% FBS to make the final suspension.

The EC pellet was suspended in the culture medium at a density of ~ 2–5 × 10^5^ cells/mL. 1 mL cell suspension was mixed with 1 mL bead suspension into a round bottom tube with a snap cap (Corning). The tube was placed in a 37 °C incubator with 5% CO_2_ for 6 h and gently shaken manually every 2 h to allow cells to evenly distribute on the beads. Manual shaking cannot be replaced by a shaker, as most cells will not adhere under continuous shaking. After 6 h of incubation, the mixture (2 mL) was transferred to a 35-mm Petri dish and incubated for 48 h until most beads were fully covered with cells. Next, the spheroid suspension was transferred to a Falcon tube and left for 5 min allowing spheroids to settle. The culture medium was aspirated carefully and the same amount (2 mL) of DMEM/F12 was added to re-suspend spheroids.

Matrigel^®^ Basement Membrane Matrix (Corning^®^) should be kept on ice. Also, pipette tips and microcentrifuge tubes used for matrix preparation should be pre-chilled. For culture preparation in a 24-well format, 440 μL DMEM and 100 μL of spheroid suspension were mixed in a sterile microcentrifuge tube. To obtain the final concentration of Matrigel^®^ (5 mg/mL), to the previously obtained spheroid suspension in DMEM/F12, 460 μL of the matrix was added (10.9 mg/mL). They were altogether carefully pipetted up and down to mix well. Four hundred microliters of the mixture was dispensed in each well of the culture plate and the plate was incubated at 37 °C for at least 30 min until a solid gel formed. After that time, 500 μL of warm (37 °C) DMEM/F12 medium with 1% FBS was added carefully along the sidewall onto the gel. Additional bFGF was added to the medium in the experimental wells in the amount of 10 ng/mL (Oswald et al 2004). Thus, the prepared culture plate was cultivated for 14 days, changing the medium to fresh every 2 days. After completion of the culture, the fixation and permeabilization procedure was performed as described in “Immunofluorescence.” Then, rhodamine-labeled phalloidin (Invitrogen™ Thermo Fisher Scientific, Waltham, MA, USA) diluted 1:10 in TBS was applied. Cells were incubated for 30 min at RT and in the dark. After incubation and washing in TBS, the plate was sealed with a DAPI-Fluoroshield mounting gel (Sigma-Aldrich) and imaged using the Olympus FLUOVIEW FV1200 laser scanning confocal microscope (Olympus, Tokyo, Japan), using laser wavelengths and powers set for respective fluorochromes as indicated by their manufacturers. Then, 3D modeling was performed on a selected series of photos of optical sections, including (i) scanning of 21 sections with a thickness of 4.45 μm each in the "*z*" axis in Imaris 7.2 software (Bitplane Oxford Instruments Company); (ii) simple 3D modeling using the "shadow projection" technique, also using Imaris software.

### Statistical analysis

Statistical analysis was performed using Statistica v.13.1 software (StatSoft, Inc., Tulsa, OK, USA). For cell culture experiments, *n* = 5 (experiments were performed in quadruplicate). The Levene's test for homogeneity of variance, the Shapiro–Wilk test for normality, and the one-way ANOVA followed by Tukey’s honestly significant difference (HSD) post hoc test were used to assess differences between control and experimental cultures. Western blot and real-time PCR were repeated three times (in duplicate). The data were expressed as the mean ± standard deviation. Statistical significance was established accordingly: *P ≤ 0.05, **P ≤ 0.01, and ***P ≤ 0.001.

## Results

### Morphology of poPSCs, protein, and gene expression of selected MSC markers

**Fig. 1:**
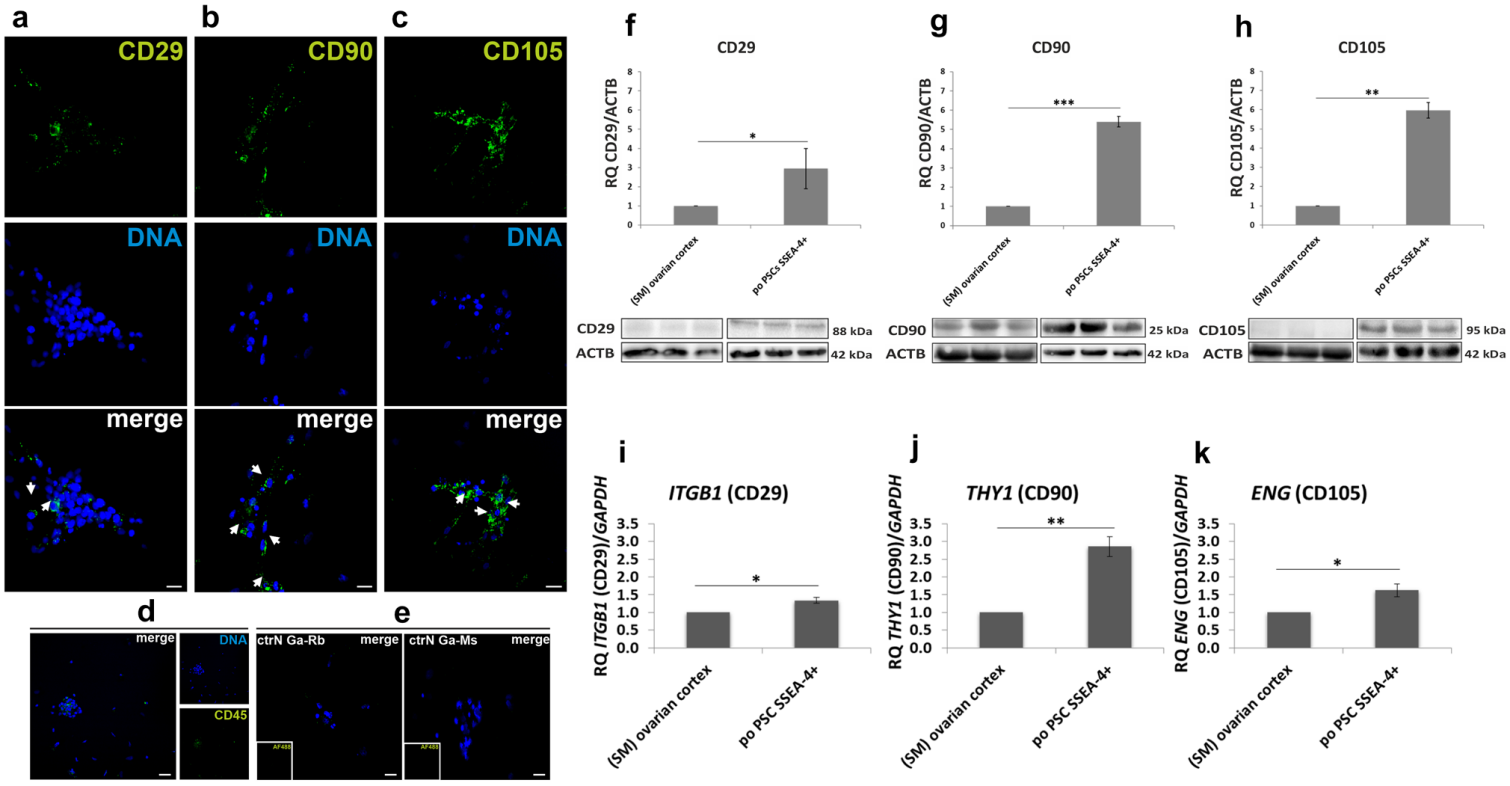
Expression of multipotent/mesenchymal stem cell markers after the third passage, as analyzed by immunofluorescence, Western blotting, and qRT-PCR. Immunofluorescence: The green signal from the fluorescent dye Alexa Fluor 488 (white arrows) indicated a specific, surface location of CD29 (**a**), CD90 (**b**), and CD105 (**c**) proteins. Analysis showed a lack of CD45 (**d**) protein (negative marker of MSCs). The nuclei were counterstained with DAPI (blue). The negative control (ctrN) for goat anti-rabbit (Ga-Rb) and goat anti-mouse (Ga-Ms) antibodies showed the lack of nonspecific staining (**e**), scale bar = 100 µm. Western blot: Expression of selected multipotent/mesenchymal stem cell markers at the total protein level in poPSC SSEA-4^+^ after the third passage compared with porcine ovarian cortex homogenate (starting material, SM, for the isolation of poPSC SSEA-4^+^). The relative expression of CD29 (**f**), CD90 (**g**), and CD105 (**h**) protein based on optical density measurements of the bands constituting a specific signal and calculated relative to the reference protein β-actin (ACTB). The results represent the mean of *n* = 5 ± standard deviation (SD). qRT-PCR: Analysis of the expression of marker genes for multipotent/mesenchymal stem cells such as *ITGB1* (**i**), *THY1* (**j**), and *ENG* (**k**) after the third passage compared with porcine ovarian cortex homogenate (starting material, SM, for the isolation of poPSC SSEA-4^+^) at the transcript level as shown by qRT-PCR. The results (2^−ΔΔCt^) are presented as the mean values with *n* = 3 ± standard deviation (SD). Statistical analysis: homogeneity of variance—Levene’s test, normality of distribution—Shapiro–Wilk test, one-way ANOVA and Tukey’s HSD post hoc test, *P < 0.05; **P < 0.01; ***P < 0.001

poPSCs after the third passage (70–80% confluence) formed a monolayer of flat fibroblast-like cells, with spindle-shaped morphology with large aggregates present among them. poPSCs showed high expression of specific MSC markers: CD29, CD90, and CD105. Importantly, poPSCs were negative for a typical lymphocytic marker: CD45 (Fig. 1). Immunofluorescence analysis for antigens typically expressed by MSCs and cell morphology confirmed that poPSCs, obtained by immunomagnetic isolation from the porcine ovarian cortex represent a true, but nonhomogeneous, MSC population. Western blot analyses confirmed data obtained from immunofluorescence. The statistically significant (**P < 0.05) higher expression of CD29, CD90, and CD105 was found in poPSC cells compared to control ovarian cortex homogenates (Fig. 1). Protein expression was also examined at the level of corresponding genes: *ITGB1*, *THY,* and *ENG*, respectively, employing quantitative real-time PCR. The *ITBG1* and *ENG* mRNA expression level in poPSCs was higher compared to the ovarian cortex (control) (*P < 0.05) with that of *THY* being almost three times higher than in the control (**P < 0.01) (Fig. 1).

### Differentiation of poPSCs into endothelial-like cells

**Fig. 2:**
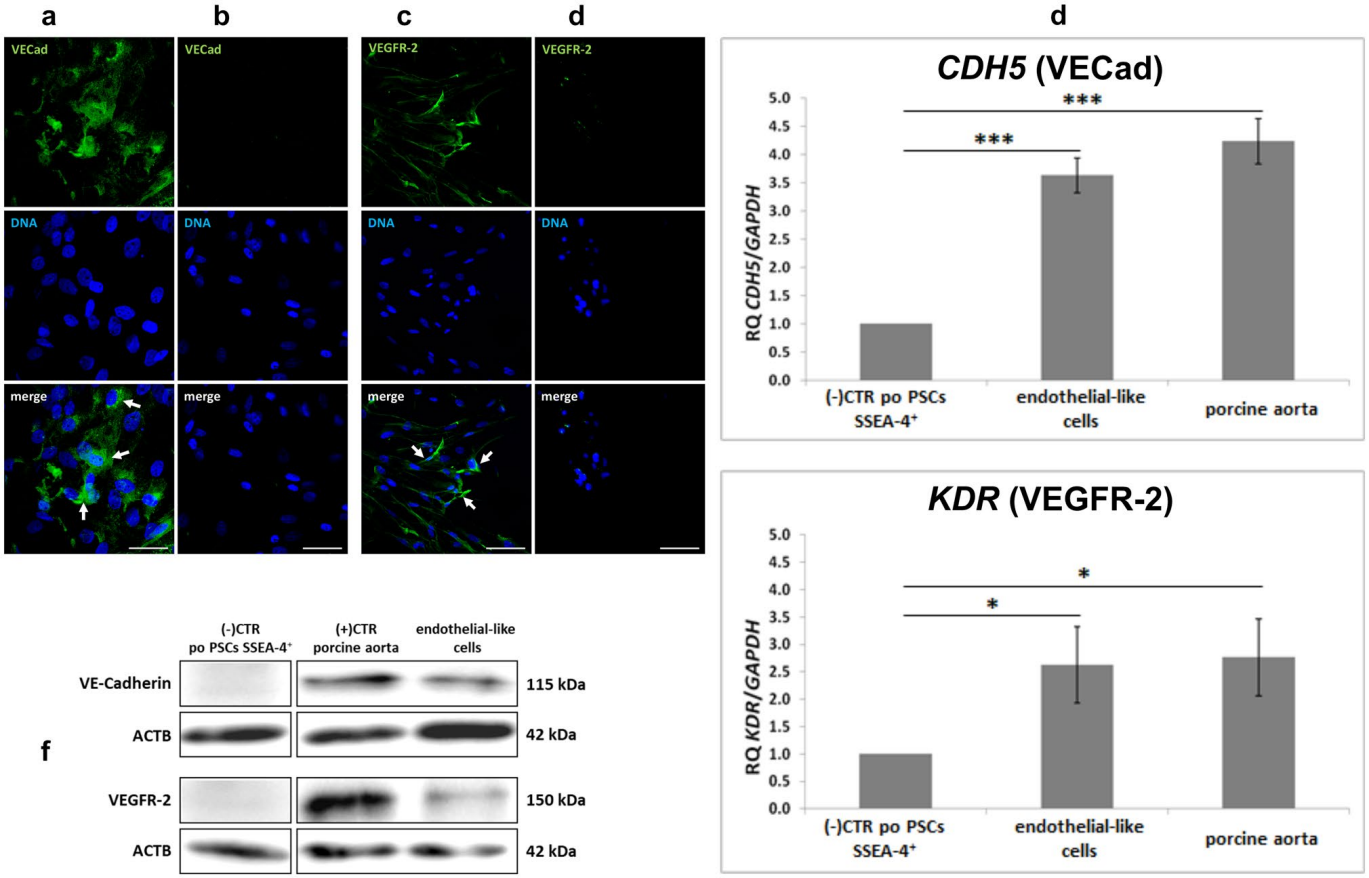
Expression of endothelial cell markers after 14 days of the differential culture and poPSCs after the third passage (negative control), as analyzed by immunofluorescence, Western blotting, and qRT-PCR: Immunofluorescence: The green signal from the fluorescent dye Alexa Fluor 488 (white arrows) indicated a specific, cytoplasmic and membrane location of VE-cadherin (**a**) and VEGFR-2 (**c**) proteins in poPSCs, differentiated into vascular endothelial cells. The lack of green signal from the fluorescent dye Alexa Fluor 488 indicated a lack of specific cytoplasmic and membrane location of VE-cadherin (**b**) protein and lack of specific membrane location of VEGFR-2 in poPSCs after the third passage. The nuclei were counterstained with DAPI (blue), scale bar (**a**, **b**) = 200 µm, (**c**,** d**) = 50 µm. Western blot: Expression of vascular endothelial cell markers (VE-cadherin and VEGFR-2) at the total protein level in poPSC SSEA-4^+^ after differentiation into vascular endothelial cell homogenate compared with poPSC SSEA-4^+^ homogenate after the third passage (negative control) and porcine aorta homogenate (positive control). The presence of the proteins sought is shown in the form of specific bands. β-actin (ACTB) was used as a loading control for each analyzed protein. qRT-PCR: Analysis of the expression of marker genes for vascular endothelial cells such as *CDH5* and *KDR* (**d**) after 14-day differentiation into vascular endothelium compared with poPSC SSEA-4^+^ after the third passage (negative control) and porcine aorta (positive control) at the transcript level as shown by qRT-PCR. The results (2^−ΔΔCt^) are presented as the mean values with *n* = 3 ± standard deviation (SD). Statistical analysis: homogeneity of variance—Levene’s test, normality of distribution—Shapiro–Wilk test, one-way ANOVA and Tukey’s HSD post hoc test, *P < 0.05; **P < 0.01; ***P < 0.001

After 14 days of PSC differentiation into ECs, immunofluorescence analysis was performed for the presence of VECad and VEGFR-2 for the basal confirmation of EC phenotype. Undifferentiated poPSCs showed no staining for these markers, but after 14 days of differentiation culture, the overall fluorescence intensity of VECad and VEGFR-2 was markedly enhanced (Fig. 2). The results of the immunofluorescence analysis are consistent with those of the WB and quantitative real-time PCR analyses.

The protein abundance of VE-cadherin and VEGFR-2 was examined by Western blot in poPSCs differentiated into vascular cells. Immunodetectable VECad protein was observed as a single band near the 150 kDa, while VEGFR-2 protein was seen as a band near the 146 kDa position of the SDS gel. Homogenates of porcine aorta fragments served as a positive control (Fig. 2). Importantly, in lysates obtained from non-differentiated poPSCs (negative control) these proteins were not found (Fig. 2). Stripped immunoblots were also used for β-actin that served as a control for equal protein loading. The bands were analyzed densitometrically, and the data obtained for each protein were normalized against its corresponding β-actin (Fig. 2).

The expression of *CDH* and *KDR* mRNA was confirmed employing quantitative real-time PCR (Fig. 2). The level of *CDH5* mRNA expression in poPSCs after 14 days of differentiation into vascular endothelium was more than three times higher than in the control sample, i.e. undifferentiated poPSCs (***P < 0.001). In the pig aorta (positive control), the level of *CDH5* mRNA expression was four times higher than in the control, and it was a statistically significant difference (***P < 0.001). There were no statistically significant differences in the level of *CDH5* mRNA expression in the aorta compared to the level of expression in obtained ECs. The level of *KDR* mRNA expression in poPSCs after 14-day differentiation into vascular endothelium was nearly three times higher than in the control of poPSCs (*P < 0.05). In the pig aorta (positive control), the level of *KDR* mRNA expression was four times higher than in the control and it was a statistically significant difference (*P < 0.05). There were no differences in the level of *KDR* mRNA expression in the aorta from that in ECs obtained. The above analysis showed that the poPSCs under the influence of the respective compounds were differentiated into the vascular endothelium, as evidenced by the comparable expression level in endothelially differentiated poPSCs to the expression level in the aorta being a positive control.

### Functional status of endothelial cells: migration and tube formation assays

**Fig. 3:**
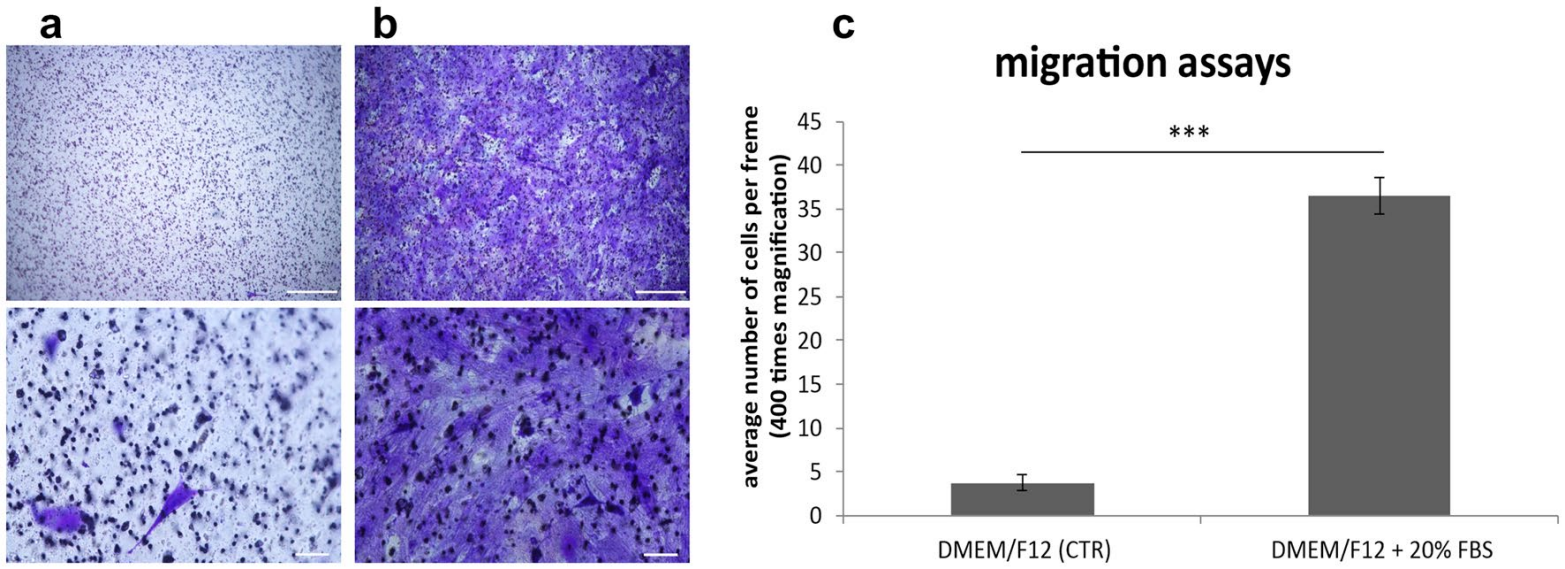
The migration test. PSCs SSEA-4^+^ differentiated into endothelial cells, migrated through 3-µm pores under the influence of serum (**b**) compared with cells in serum-free medium (**a**) crystal violet staining after 48 h of the experiment. The microphotographs showed a representative staining result from one of the repetitions, scale bar (upper **a**, **b**) = 100 µm, (lower **a**, **b**) = 20 µm. The results represent the mean of *n* = 5 ± standard deviation (SD). Statistical analysis: homogeneity of variance—Levene’s test, normality of distribution—Shapiro–Wilk test, one-way ANOVA and Tukey’s post hoc test, *P < 0.05; **P < 0.01; ***P < 0.001 (**c**)

The migration test performed after 14 days of poPSC differentiation into ECs showed that the vast majority of them migrated through the 3-µm filter towards the chemoattractant, FBS, whereas in the control experiments, only single cells were observed on the lower surface of the filter (Fig. 3). The migration analysis proved that the ECs, obtained by differentiation from poPSCs, are capable of positive chemotaxis. Moreover, the ability of these cells to pass through pores as small as 3 µm in diameter suggests that we are indeed dealing with ECs.

**Fig. 4:**
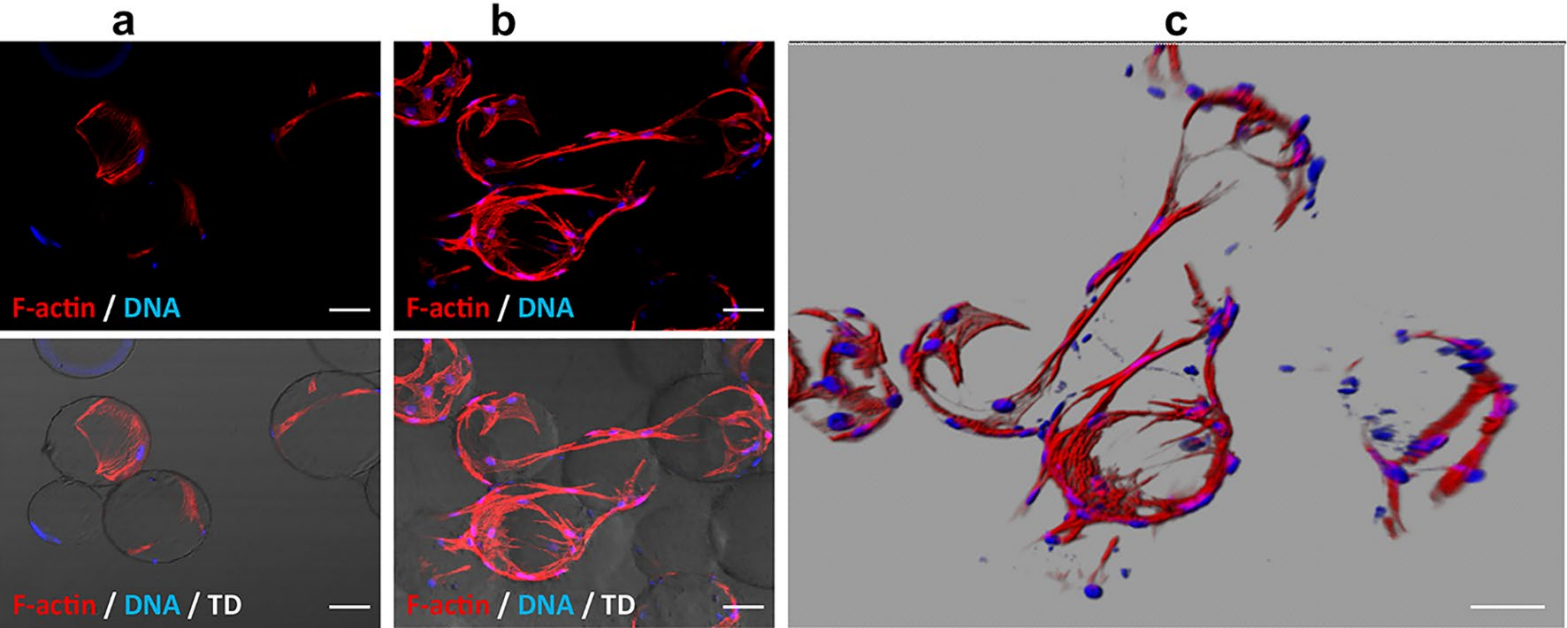
Tube formation assays. PSCs SSEA-4^+^ differentiated into the endothelium in the tube (pre-vessel) formation assay under the influence of bFGF 14 days after test initiation (**b**) compared to poPSCs after the third passage (negative control, **a**). Red signal, F-actin visualized with Rhodamine B-conjugated phalloidin. The blue signal indicates DAPI, which stains the DNA of cell nuclei. In the photos at the bottom of figure, transmitted light (TD) was turned on to show the dextran microcarrier beads. In the image **c** 3D modeling using the "shadow projection" technique. Scanning in the "*z*" axis, 21 cross-sections with a thickness of 4.45 μm each; assembly: Imaris 7.2 software (Bitplane Oxford Instruments Company), scale bar = 100 µm

During the 14 days of EC culture using the microcarrier-based spheroids in the culture medium supplemented with bFGF, cells started to branch off already after 7 days, and formed tubes were visible on day 14. Interestingly, ECs growing without the addition of bFGF, were incapable of forming the tubes. ECs were grown on the surface of the beads and did not create communication links with each other (Fig. 4). The 3D modeling technique used to show spatial networks of communicating vascular ECs confirmed that poPSCs differentiating into the vascular endothelium can organize themselves into spatial systems under the influence of growth factors. This is important because the ability of ECs to create such structures is necessary for the initiation of angiogenesis.

## Discussion

According to a significant number of recent studies, ASCs are self-renewing, multipotent progenitor cells, present virtually in all tissues during most stages of development. Since they possess trans-differentiation potential, their main role is in the renewal and repair of aged or damaged tissue. Consequently, ASCs attract much attention from biotechnologists and clinicians (Mimeault et al. 2007; Mahla 2016). Successful isolation and in vitro maintenance of ASCs is of increasing importance in the field of applied biology. Our team's previous results (Wartalski et al. 2016, 2020) combined with data from other laboratories (Li and Clevers 2010; Bhartiya 2015; Parte et al. 2015; Esmaeilian et al. 2017) indicate that much progress has been made in the field of ovarian stem cells in mammals. Interestingly, the greatest controversy concerns the plasticity of stem cells isolated from the human ovarian cortex. Although earlier research results suggested that human ovarian stem cells have the potential to differentiate in vitro into oocytes (Virant-Klun et al. 2009), the latest ones indicate that there are no germline stem cells in the adult human ovarian cortex (Wagner et al. 2020). In turn, poPSCs isolated from the porcine ovarian cortex express pluripotency markers and can differentiate into various cell types, as demonstrated by our successful attempts to differentiate them into cells of the nervous system such as neurons and glial cells (Wartalski et al. 2020). Moreover, many compounds can promote their differentiation. The plasticity of that population of ovarian cells offers great opportunity; however, their full phenotypic characterization is necessary, along with a complete understanding of all possible differentiation pathways and the mechanisms controlling the course of these phenomena. This work is, therefore, another attempt to better describe their distinctive nature.

This study demonstrated for the first time the specific cellular distribution of mesenchymal stem cell multipotency markers in poPSCs isolated immunomagnetically from the ovarian cortex of postnatal pigs. Immunofluorescence analysis clearly showed the specific surface localization of the markers CD29, CD90, and CD105. The lack of CD45 antigen was also confirmed by immunofluorescence. Western blot analysis not only confirmed the presence of all three tested markers in poPSCs, but also showed their increased expression compared to ovarian cortex tissue. Similarly, high expression of all three MSC markers (*ITGB1, THY1, ENG*) was observed at the transcript level. The obtained results allow us to postulate that poPSCs constitute a subpopulation of MSCs in the ovary and as such are characterized by high differentiation potential.

Bearing in mind the accumulating evidence indicating both morphological and functional heterogeneity in MSC populations during in vitro expansion, even within single colonies (Tremain et al. 2001; Rennerfeldt and Vliet 2016; Rennerfeldt et al. 2019), it seems possible that the population of ovarian MSCs we isolated was not homogeneous. Instead it might have contained multiple subpopulations, and this will require further characterization in future research.

The finding that stem cells isolated from the ovarian cortex are MSCs is relatively new. This study is the first to characterize porcine ovarian stem cells as MSCs. Hill et al. (2018) established MSC lines derived from canine ovarian tissue based on their ability to rapidly (the first 3 h after plating) adhere to plastic culture dishes. They characterized them in terms of molecular markers presence (CD90 and CD44) and differentiation capacity. They concluded that both morphological and molecular properties of MSCs isolated from the canine ovary were similar to adipose-derived MSCs. Our results are consistent with those reported by them. Similar to canine ovarian-derived MSCs, those isolated from the porcine ovary are morphologically similar to fibroblasts, form colonies, and show rapid proliferation. To validate novel MSC populations of cells, they should adhere to plastic and show the presence/absence of specific surface markers and differentiation properties (Dominici et al. 2006). Both porcine and canine ovarian-derived MSCs showed the presence of several markers identified in adipose tissues, such as CD105, CD90, and CD44 (our team unpublished data). This suggests similarities between MSCs originating from the ovary and those from the adipose tissue. In addition, neither ovarian- nor adipose-derived MSCs possess hematopoietic markers such as CD45. The expression of mesenchymal markers in the ovary may be associated with the involvement of these cells in tissue repair after ovulation (Ahmed et al. 2007). This process of wounding and resulting repair throughout the female reproductive life confers plasticity to the ovary and promotes the expression of both epithelial- and mesenchymal-specific genes needed for tissue remodeling. CD90 plays also a role in intercellular adhesion (Saalbach et al. 2000). Interestingly it is also expressed by porcine theca stem cells, which showed the ability to differentiate into osteocytes and adipocytes (Lee et al. 2013). In the mouse, CD90 was dominantly expressed in somatic compartments of ovarian follicles, suggesting that it might play different functions (Tepekoy et al. 2015).

Many clinical studies confirmed that MSCs can differentiate into a variety of cell types (Sacchetti et al. 2016; Galipeau and Sensébé 2018), including ECs. Based on the above, stem cell-based therapies have been proposed to support endothelial regeneration (De Luca al. 2019). Because the vascular endothelium consists of small, flat cells with a mesenchymal shape, it is therefore not surprising that MSCs can easily differentiate into ECs. Indeed, the efficiency of poPSC differentiation into vascular endothelium was much higher than previously carried out neural differentiation of the same cells (Wartalski et al. 2020). poPSCs under the influence of the endothelial differentiation promoting factors formed a nonhomogeneous monolayer of ECs within 14 days. For comparison, a 14-day culture differentiating of poPSCs into cells of the nervous system resulted in obtaining a less numerous, heterogeneous population of neurons and glial cells.

The relative ease of MSC differentiation into the vascular endothelium might also be explained by one of the theories about the perivascular origin of MSCs (Crisan et al. 2008, 2012). Perivascular cells, mainly pericytes, appear to be present in many organs and tissues, including adipose tissue, skeletal muscle, pancreas, and placenta. Moreover, perivascular cells maintained in long-term in vitro culture showed the expression of MSC markers and the ability for chemotaxis. Based on these multipotent capabilities, perivascular cells/pericytes are considered very similar to MSCs (MSC-like) (Bara et al. 2014).

The present research reported that after 14-days of poPSC differentiation into ECs, they showed the expression of key endothelial markers: VE-cadherin and VEGFR-2 both at the protein level (immunofluorescence and WB analysis) and corresponding genes (qRT-PCR; *CDH5, KDR*). Expression levels of both these genes were comparable to those found in the aorta samples and much higher than in undifferentiated poPSCs, which confirms successful differentiation of poPSCs into ECs. Differentiation of various MSCs into the endothelium has been successfully carried out for over 10 years. Oswald et al. (2004) isolated MSCs from human bone marrow. The obtained cells expressed typical MSC markers such as CD44, CD73, CD90, and CD105 and at the same time showed no hematopoietic and EC markers. Such MSCs were differentiated in the presence of 50 ng/mL VEGF and 2% serum concentration in the culture medium. Already such a relatively low stimulation with one growth factor only and a low amount of serum was enough to differentiate the bone marrow-derived MSCs into the vascular endothelium. The analyzes carried out by the flow cytometry showed that the above-mentioned cells strongly express such endothelial markers as FLT-1 (VEGF type 1 receptor) and VEGFR-3. In turn, the immunofluorescence analysis confirmed the presence and localization of the von Willebrand factor, an essential blood component that determines normal platelet adhesion and aggregation and is produced by ECs. Additionally, in an in vitro angiogenesis test, MSCs plated on an artificial basement membrane matrix (Matrigel^®^) in the presence of VEGF began to form elongated, tubular structures resembling primitive capillaries (Oswald et al. 2004). In this study, a very similar tube formation assay has been performed. However, endothelial differentiated poPSC cells have already been used for seeding on microcarrier-based spheroids. When the cells adhered to the beads and started to grow on their surface, the beads were placed in an artificial basement membrane matrix (Matrigel^®^). Fourteen days of culture in the presence of bFGF resulted in the formation of elongated, tubular structures resembling primitive capillaries. Pankajakshan et al. (2013) were the first to demonstrate using functional assays and mRNA and protein expression analysis of ECs markers (VE-cadherin, PECAM-1, VEGF-R1, and VEGF-R2) that porcine bone marrow-derived MSCs have the potential to differentiate into ECs in the presence of endothelial growth supplements and VEGF in vitro. The cultured bone marrow-derived cells were CD34^–^, CD44^+^, CD90^+^ and showed mesodermal lineage differentiation. Additionally, these cells showed profuse sprouting of capillary tubes and closed polygon formation in the angiogenesis assay. Similar results were obtained in the current study. In other studies (Silva et al. 2005), the ability of canine MSCs to differentiate in vivo has been demonstrated. Moreover, not only can bone marrow MSCs be efficiently differentiated into vascular endothelium, but an important group of MSCs are cells derived from the amniotic fluid. Human MSCs can be obtained from the amniotic fluid by amniocentesis during the second trimester of pregnancy. Such cells show MSC markers such as CD44, CD73, CD90, and human leukocyte antigen—ABC (HLA-ABC), which was confirmed by flow cytometry. At the same time, MSCs isolated from the amniotic fluid are negative for markers such as CD31, CD34, CD45, CD117, and HLA-DR. MSCs from the amniotic fluid were differentiated into ECs using VEGF culture medium supplementation and then analyzed for the expression of endothelial-specific markers and functions (RT-qPCR and immunofluorescence methods). These analyzes showed that MSCs differentiated into the vascular endothelium exhibit von Willebrand factor, VEGFR-2, CD31, and endothelial nitric oxide synthase (eNOS).

To finally confirm the differentiation of poPSCs into the vascular endothelium, their ability to migrate through the porous membrane under the influence of a chemoattractant was checked. This is a fairly common procedure known for many years as the Boyden chamber test. Previously it was used to test the ability of vascular ECs to migrate through an 8-μm pore size membrane under the influence of VEGF as a chemoattractant (Yoshida et al. 1996; Kramer et al. 2013). In the present study, the Boyden test was modified using a 3-µm membrane and medium with 20% FBS as a chemoattractant. After 48 h, sevenfold greater migration was observed in the chemoattractant trials as compared to the FBS-free control. The rate of migration through such small pores, with the presence of endothelial markers such as VE-cadherin and VEGFR-2 indicate that cells obtained by differentiation from poPSCs are indeed vascular ECs. Since EC heterogeneity has been described at the level of cell morphology, function, gene expression, and antigen composition (Aird 2007a, b, 2012), future studies will be needed to more precisely characterize the poPSC-derived endothelial-like cells.

## Conclusions

In summary, poPSCs isolated from the postnatal ovarian cortex of sexually immature pigs, are multipotent. They display numerous surface markers of mesenchymal stem cells (including CD29, CD90, CD105) and therefore can be considered as a heterogeneous population of MSCs in the ovary. poPSCs escape the rigid classification framework because we successfully differentiated them into cells originated from the two germ layers: various cells of the nervous system and now the vascular ECs. The plasticity of the isolated population of cells offers a great opportunity. On the other hand, of concern is the ease with which poPSCs differentiate into vascular endothelium, since it may be related to their possible role in initiating angiogenesis in ovarian tumors. Porcine ovarian tissue is accessible and has high plasticity, holding promise for applications in regenerative medicine. However, xenogeneic transplantation from pigs to humans is compromised by high immune incompatibility and a complex rejection process. However, the rapid development of genetic engineering techniques enables genome modifications in pigs that reduce the cross-species immune barrier (Ryczek et al. 2021). Xenotransplantation is a multidisciplinary undertaking, requiring the development of a range of research methods. In recent years, advances have been made in areas of developing and introducing the appropriate gene constructs, determining the characteristics of the transgenic animals produced, or ensuring donor and recipient histocompatibility. All this in terms of both knowledge and technology, may bring the successful application of xenotransplantation closer to reality. Given the fact that genetically modified pigs can become cell or tissue donors, following the phenotypic characterization of poPSCs, the next challenge would be to investigate the different differentiation pathways of poPSCs and the mechanisms that control these phenomena.

## Data Availability

All data generated or analyzed during this study are included in this published article. Requests for material should be made to the corresponding author.

## References

[CR1] Abbott NJ, Rönnbäck L, Hansson E (2006). Astrocyte-endothelial interactions at the blood-brain barrier. Nat Rev Neurosci.

[CR2] Ahmed N, Thompson EW, Quinn MA (2007). Epithelial-mesenchymal interconversions in normal ovarian surface epithelium and ovarian carcinomas: an exception to the norm. J Cell Physiol.

[CR3] Aird WC (2007). Phenotypic heterogeneity of the endothelium: I. Structure, function, and mechanisms. Circ Res.

[CR4] Aird WC (2007). Phenotypic heterogeneity of the endothelium: II. Representative vascular Beds. Circ Res.

[CR5] Aird WC (2012). Endothelial cell heterogeneity. Cold Spring Harb Perspect Med.

[CR6] Araña M, Mazo M, Aranda P, Pelacho B, Prosper F (2013). Adipose tissue-derived mesenchymal stem cells: isolation, expansion, and characterization. Methods Mol Biol Clifton NJ.

[CR7] Baksh D, Song L, Tuan RS (2004). Adult mesenchymal stem cells: characterization, differentiation, and application in cell and gene therapy. J Cell Mol Med.

[CR8] Bara JJ, Richards RG, Alini M, Stoddart MJ (2014). Concise review: bone marrow-derived mesenchymal stem cells change phenotype following in vitro culture: Implications for basic research and the clinic. Stem Cells.

[CR9] Bhartiya D (2015). Ovarian stem cells are always accompanied by very small embryonic-like stem cells in adult mammalian ovary. J Ovarian Res.

[CR10] Bianco P, Cao X, Frenette PS, Mao JJ, Robey PG, Simmons PJ, Wang CY (2013). The meaning, the sense and the significance: translating the science of mesenchymal stem cells into medicine. Nat Med.

[CR11] Bordy R, Totoson P, Prati C, Marie C, Wendling D, Demougeot C (2018). Microvascular endothelial dysfunction in rheumatoid arthritis. Nat Rev Rheumatol.

[CR12] Bradford MM (1976). A rapid and sensitive method for the quantitation of microgram quantities of protein utilizing the principle of protein-dye binding. Anal Biochem.

[CR13] Burian B, Probst F, Palla B, Riedel C, Saller MM, Cornelsen M, Konig F, Schieker M, Otto S (2017). Effect of hypoxia on the proliferation of porcine bone marrow-derived mesenchymal stem cells and adipose-derived mesenchymal stem cells in 2- and 3-dimensional culture. J Craniomaxillofac Surg.

[CR14] Catacchio I, Berardi S, Reale A, De Luisi A, Racanelli V, Vacca A, Ria R (2013). Evidence for bone marrow adult stem cell plasticity: properties, molecular mechanisms, negative aspects, and clinical applications of hematopoietic and mesenchymal stem cells transdifferentiation. Stem Cells Int.

[CR15] Ceusters J, Lejeune J-P, Sanderson C, Niesten A, Lagneaux L, Serteyn D (2017). From skeletal muscle to stem cells: an innovative minimally-invasive process for multiple species. Sci Rep.

[CR16] Corada M, Liao F, Lindgren M, Lampugnani MG, Breviario F, Frank R, Muller WA, Hicklin DJ, Bohlen P, Dejana E (2001). Monoclonal antibodies directed to different regions of vascular endothelial cadherin extracellular domain affect adhesion and clustering of the protein and modulate endothelial permeability. Blood.

[CR17] Crisan M, Yap S, Casteilla L, Chen CW, Corselli M, Park TS, Peault B (2008). A perivascular origin for mesenchymal stem cells in multiple human organs. Cell Stem Cell.

[CR18] Crisan M, Corselli M, Chen WC, Peault B (2012). Perivascular cells for regenerative medicine. J Cell Mol Med.

[CR19] De Luca M, Aiuti A, Cossu G, Parmar M, Pellegrini G, Robey PG (2019). Advances in stem cell research and therapeutic development. Nat Cell Biol.

[CR20] D'Ippolito G, Diabira S, Howard GA, Menei P, Roos BA, Schiller PC (2004). Marrow-isolated adult multilineage inducible (MIAMI) cells, a unique population of postnatal young and old human cells with extensive expansion and differentiation potential. J Cell Sci.

[CR21] Dominici M, Le Blanc K, Mueller I, Slaper-Cortenbach I, Marini F (2006). Minimal criteria for defining multipotent mesenchymal stromal cells. The International Society for Cellular Therapy position statement. Cytotherapy.

[CR22] Dudley AC (2012). Tumor endothelial cells. Cold Spring Harb Perspect Med.

[CR23] Esmaeilian Y, Atalay A, Erdemli E (2017). Putative germline and pluripotent stem cells in adult mouse ovary and their in vitro differentiation potential into oocyte-like and somatic cells. Zygote.

[CR24] Galipeau J, Sensébé L (2018). Mesenchymal stromal cells: clinical challenges and therapeutic opportunities. Cell Stem Cell.

[CR25] Galliot B, Ghila L (2010). Cell plasticity in homeostasis and regeneration. Mol Reprod Dev.

[CR26] Gorczyca G, Wartalski K, Tabarowski Z, Duda M (2019). Effects of vinclozolin exposure on the expression and activity of SIRT1 and SIRT6 in the porcine ovary. J Physiol Pharmacol.

[CR27] Grompe M (2002). Adult versus embryonic stem cells: it's still a tie. Mol Ther.

[CR28] Herbst RS (2004). Review of epidermal growth factor receptor biology. Int J Radiat Oncol Biol Phys.

[CR29] Hill AB, Hill JE, Bressan FF, Miglino MA, Garcia JM (2018). Derivation and differentiation of canine ovarian mesenchymal stem cells. J vis Exp.

[CR30] Hollenberg MD, Gregory H (1980). Epidermal growth factor-urogastrone: biological activity and receptor binding of derivatives. Mol Pharmacol.

[CR31] Holmes K, Roberts OL, Thomas AM, Cross MJ (2007). Vascular endothelial growth factor receptor-2: structure, function, intracellular signalling and therapeutic inhibition. Cell Signal.

[CR32] Hryhorowicz M, Zeyland J, Słomski R, Lipiński D (2017). Genetically modified pigs as organ donors for xenotransplantation. Mol Biotechnol.

[CR33] Ikhapoh IA, Pelham CJ, Agrawal DK (2015). Sry-type HMG box 18 contributes to the differentiation of bone marrow-derived mesenchymal stem cells to endothelial cells. Differentiation.

[CR34] Jiang Y, Jahagirdar BN, Reinhardt RL, Schwartz RE, Keene CD, Ortiz-Gonzalez XR, Du J, Aldrich S, Lisberg A, Low WC, Largaespada DA, Verfaillie CM (2002). Pluripotency of mesenchymal stem cells derived from adult marrow. Nature.

[CR35] Juin P, Hueber AO, Littlewood T, Evan G (1999). c-Myc-induced sensitization to apoptosis is mediated through cytochrome c release. Genes Dev.

[CR36] Justus CR, Leffler N, Ruiz-Echevarria M, Yang LV (2014). In vitro cell migration and invasion assays. J vis Exp.

[CR37] Kern S, Eichler H, Stoeve J, Kluter H, Bieback K (2006). Comparative analysis of mesenchymal stem cells from bone marrow, umbilical cord blood, or adipose tissue. Stem Cell.

[CR38] Koizumi K, Wang G, Park L (2016). Endothelial dysfunction and amyloid-β-induced neurovascular alterations. Cell Mol Neurobiol.

[CR39] Kramer N, Walzl A, Unger C, Rosner M, Krupitza G, Hengstschläger M, Dolznig H (2013). In vitro cell migration and invasion assays. Mutat Res.

[CR40] Laemmli UK (1970). Cleavage of structural proteins during the assembly of the head of bacteriophage T4. Nature.

[CR41] Lee YM, Kumar BM, Lee JH, Lee WJ, Kim TH, Lee SL, Rho GJ (2013). Characterisation and differentiation of porcine ovarian theca-derived multipotent stem cells. Vet J.

[CR42] Li L, Clevers H (2010). Coexistence of quiescent and active adult stem cells in mammals. Science.

[CR43] Livak KJ, Schmittgen TD (2001). Analysis of relative gene expression data using real-time quantitative PCR and the 2^−^^∆∆Ct^ method. Methods.

[CR44] Mahla RS (2016). Stem cells applications in regenerative medicine and disease therapeutics. Int J Cell Biol.

[CR45] Mimeault M, Hauke R, Batra SK (2007). Stem cells: a revolution in therapeutics—recent advances in stem cell biology and their therapeutic applications in regenerative medicine and cancer therapies. Clin Pharmacol Ther.

[CR46] Morrison S, Spradling AC (2008). Stem cells and niches: mechanisms that promote stem cell maintenance throughout life. Cell.

[CR47] Nakatsu MN, Hughes CC (2008). An optimized three-dimensional in vitro model for the analysis of angiogenesis. Methods Enzymol.

[CR48] NIH Stem Cell Information Home Page. In Stem Cell Information. Bethesda, MD: National Institutes of Health, US Department of Health and Human Services, 2016 https://stemcells.nih.gov/info/2001report/chapter4.htm

[CR49] Oswald J, Boxberger S, Jorgensen B, Feldman S, Ehninger B, Bornhauser M, Werner C (2004). Mesenchymal stem cells can de differentiated into endothelial cells in vitro. Stem Cells.

[CR50] Pankajakshan D, Kansal V, Agrawal DK (2013). In vitro differentiation of bone marrow derived porcine mesenchymal stem cells to endothelial cells. J Tissue Eng Reg Med.

[CR51] Parte S, Patel H, Sriraman K, Bhartiya D (2015). Isolation and characterization of stem cells in the adult mammalian ovary. Methods Mol Biol.

[CR52] Patel H, Bhartiya D, Parte S (2018). Further characterization of adult sheep ovarian stem cells and their involvement in neo-oogenesis and follicle assembly. J Ovarian Res.

[CR53] Peruzzi F, Prisco M, Dews M, Salomoni P, Grassilli E, Romano G, Calabretta B, Baserga R (1999). Multiple signaling pathways of the insulin-like growth factor 1 receptor in protection from apoptosis. Mol Cell Biol.

[CR54] Pittenger MF, Mackay AM, Beck SC, Jaiswal RK, Douglas R, Mosca JD, Marshak DR (1999). Multilineage potential of adult human mesenchymal stem cells. Science.

[CR55] Quan R, Du W, Zheng X, Xu S, Li Q, Ji X, Yang D (2017). VEGF165 induces differentiation of hair follicle stem cells into endothelial cells and plays a role in in vivo angiogenesis. J Cell Mol Med.

[CR56] Raff M (2003). Adult stem cell plasticity: fact or artifact?. Annu Rev Cell Dev Biol.

[CR57] Rennerfeldt DA, Van Vliet KJ (2016). Concise review: when colonies are not clones: evidence and implications of intracolony heterogeneity in Mesenchymal Stem Cells. Stem Cells.

[CR58] Rennerfeldt DA, Raminhos JS, Leff SM, Manning P, Van Vliet KJ (2019). Emergent heterogeneity in putative mesenchymal stem cell colonies: single-cell time lapsed analysis. PLoS ONE.

[CR59] Ryczek N, Hryhorowicz M, Zeyland J, Lipiński D, Słomski R (2021). CRISPR/Cas technology in pig-to-human xenotransplantation research. Int J Mol Sci.

[CR60] Saalbach A, Haustein UF, Anderegg U (2000). A ligand of human thy-1 is localized on polymorphonuclear leukocytes and monocytes and mediates the binding to activated thy-1-positive microvascular endothelial cells and fibroblasts. J Invest Dermatol.

[CR61] Sacchetti B, Funari A, Remoli C, Giannicola G, Kogler G, Liedtke S, Bianco P (2016). No identical “mesenchymal stem cells” at different times and sites: human committed progenitors of distinct origin and differentiation potential are incorporated as adventitial cells in microvessels. Stem Cell Rep.

[CR62] Silva GV, Litovsky S, Assad JA, Sousa AL, Martin BJ, Vela D, Coulter SC, Lin J, Ober J, Vaughn WK, Branco RVC, Oliveira EM, He R, Geng YJ, Willerson JT, Perin EC (2005). Mesenchymal stem cells differentiate into an endothelial phenotype, enhance vascular density, and improve heart function in a canine chronic ischemia model. Circulation.

[CR63] Smith NR, Baker D, James NH, Ratcliffe K, Jenkins M, Ashton SE, Sproat G, Swann R, Gray N, Ryan A, Jurgensmeier JM, Womack C (2010). Vascular endothelial growth factor receptors VEGFR-2 and VEGFR-3 are localized primarily to the vasculature in human primary solid cancers. Clin Cancer Res.

[CR64] Soleimani M, Nadri S (2009). A protocol for isolation and culture of mesenchymal stem cells from mouse bone marrow. Nat Protoc.

[CR65] Stimpfel M, Cerkovnik P, Novakovic S, Maver A, Virant-Klun I (2014). Putative mesenchymal stem cells isolated from adult human ovaries. J Assist Reprod Genet.

[CR67] Takahashi H, Shibuya M (2005). The vascular endothelial growth factor (VEGF)/VEGF receptor system and its role under physiological and pathological conditions. Clin Sci.

[CR68] Tancharoen W, Aungsuchawan S, Pothacharoen P, Markmee R, Narakornsak S, Kieodee J, Tasuya W (2017). Differentiation of mesenchymal stem cells from human amniotic fluid to vascular endothelial cells. Acta Histochem.

[CR69] Tepekoy F, Ozturk S, Sozen B, Ozay RS, Akkoyunlu G, Demir N (2015). CD90 and CD105 expression in the mouse ovary and testis at different stages of postnatal development. Reprod Biol.

[CR70] Tremain N, Korkko J, Ibberson D, Kopen GC, DiGirolamo C, Phinney DG (2001). MicroSAGE analysis of 2353 expressed genes in a single cell-derived colony of undifferentiated human mesenchymal stem cells reveals mRNAs of multiple cell lineages. Stem Cells.

[CR71] Vanhoutte PM, Shimokawa H, Félétou M, Tang EH (2017). Endothelial dysfunction and vascular disease—a 30th anniversary update. Acta Physiol.

[CR72] Venturi S, Venturi M (2009). Iodine in evolution of salivary glands and in oral health. Nutr Health.

[CR73] Vestweber D (2008). VE-Cadherin; the major endothelial adhesion molecule controlling cellular junctions and blood vessel formation. Arterioscler Thromb Vasc Biol.

[CR74] Virant-Klun I, Rožman P, Cvjeticanin B, Vrtacnik-Bokal E, Novakovic S, Rülicke T, Dovc P, Meden-Vrtovec H (2009). Parthenogenetic embryo-like structures in the human ovarian surface epithelium cell culture in postmenopausal women with no naturally present follicles and oocytes. Stem Cells Dev.

[CR75] Wagers AJ, Weissman IL (2004). Plasticity of adult stem cells. Cell.

[CR76] Wagner M, Yoshihara M, Douagi I, Damdimopoulos A, Panula S, Petropoulos S, Lu H, Pettersson K, Palm K, Katayama S, Hovatta O, Kere J, Lanner F, Damdimopoulou P (2020). Single-cell analysis of human ovarian cortex identifies distinct cell populations but no oogonial stem cells. Nat Commun.

[CR77] Wartalski K, Tabarowski Z, Duda M (2016). Magnetic isolation and characterization of porcine ovarian putative stem cells (PSCs): an in vitro study. JFIV Reprod Med Genet.

[CR78] Wartalski K, Gorczyca G, Wiater J, Tabarowski Z, Palus-Chramiec K, Setkowicz Z, Duda M (2020). Efficient generation of neural-like cells from porcine ovarian putative stem cells–morphological characterization and evaluation of their electrophysiological properties. Theriogenology.

[CR79] Weissman IL (2000). Stem cells: units of development, units of regeneration, and units in evolution. Cell.

[CR80] Whyte JJ, Prather RS (2011). Genetic modifications of pigs for medicine and agriculture. Mol Reprod Dev.

[CR81] Wiater J, Niedziela M, Posmysz A, Wartalski K, Gajda B, Smorąg Z, Karasiński J (2018). Identification of perivascular and stromal mesenchymal stem/progenitor cells in porcine endometrium. Reprod Domest Anim.

[CR82] Yazdekhasti H, Hosseini MA, Rajabi Z, Parvari P, Salehnia M, Koruji M, Izadyar F, Aliakbari F, Abbasi M (2017). Improved isolation, proliferation, and differentiation capacity of mouse ovarian putative stem cells. Cell Reprogram.

[CR83] Yoshida A, Anand-Apte B, Zetter BR (1996). Differential endothelial migration and proliferation to basic fibroblast growth factor and vascular endothelial growth factor. Growth Factors.

